# Association between occupational exposures and sarcoma incidence and mortality: systematic review and meta-analysis

**DOI:** 10.1186/s13643-021-01769-4

**Published:** 2021-08-13

**Authors:** D. Edwards, A. Voronina, K. Attwood, A. Grand’Maison

**Affiliations:** 1 State University of New York At Buffalo, Department of Medicine, 12 Capen Hall, Buffalo, NY 14260 USA; 2 New York- Presbyterian Queens, Department of Medicine, 56-45 Main St, Flushing, NY 11355 USA; 3 Department of Biostatistics, Roswell Park Comprehensive Cancer Center, 665 Elm Street, Buffalo, NY 14203 USA; 4 Department of Sarcoma Medical Oncology, Roswell Park Comprehensive Cancer Center, 665 Elm Street, Buffalo, NY 14203 USA

**Keywords:** Sarcoma, Soft-tissue sarcoma, Epidemiology, Occupational exposure, Pesticide, Phenoxy herbicide, Chlorophenols, Agent Orange, Dioxin, Vinyl chloride monomers

## Abstract

**Background:**

Sarcomas are a rare and heterogeneous group of tumors originating from mesenchymal or connective tissue. They represent less than 1% of all adult cancers. The etiology and epidemiology of sarcomas remain understudied and poorly understood. The main objective of our study was to systematically assess the association between various occupational exposures and risk of sarcomas.

**Methods:**

We performed a systematic literature search using the PubMed, Scopus, EMBASE and Cochrane databases to identify relevant cohort and case–control studies. A meta-analysis method was applied on the incidence and mortality outcomes where the estimate with 95% confidence interval (CI) was obtained.

**Results:**

We included a total of 50 publications in our systematic review and 35 in meta-analysis. For exposures to phenoxy herbicides and chlorophenols, the pooled odds ratio (OR) for sarcoma was 1.85 (95% CI: 1.22, 2.82), based on 16 studies with 2254 participants, while the pooled standardized mortality ratio was 40.93 (95% CI 2.19, 765.90), based on 4 cohort studies with 59,289 participants. For exposure to vinyl chloride monomers the pooled risk ratios for angiosarcoma of the liver and other STS were 19.23 (95% CI 2.03, 182.46) and 2.23 (95 CI 1.55, 3.22) respectively based on 3 cohort studies with 12,816 participants. Exposure to dioxins was associated with an increased STS mortality; the pooled standardized mortality ratio was 2.56 (95% CI 1.60, 4.10) based on 4 cohort studies with 30,797 participants. Finally, woodworking occupation was associated with an increased risk of STS with the pooled OR of 2.16 (95% CI 1.39, 3.36).

**Conclusions:**

Our findings suggest a positive association between higher exposure to dioxins and increased mortality from STS, between cumulative exposure to vinyl chloride monomers and increased mortality from angiosarcoma of the liver and STS, and between woodworking occupation and STS incidence. These findings were all statistically significant.

**Supplementary Information:**

The online version contains supplementary material available at 10.1186/s13643-021-01769-4.

## Background

Sarcomas constitute a rare and heterogeneous group of tumors with over 70 different subtypes, originating from connective and mesenchymal tissue. They represent less than 1% of all adult cancers [1]. Due to the complexity and rarity of sarcomas, their etiology and epidemiology remain poorly understood [2].

Exposures to pesticides are amongst one of the most extensively studied exposures in epidemiological studies. Considerable attention has been focused on potential health effects of agricultural and non-agricultural exposures to phenoxy herbicides and chlorophenols as excess incidence and mortality for some specific cancers, including soft tissue sarcomas (STS), has been observed in exposed workers. The association between these compounds and STS was first reported in the Swedish observational study by Hardell et al. in 1977 [3]. The association was then supported by other Swedish case–control studies conducted in the 1970s and 1980s. A meta-analysis of four Swedish case–control studies confirmed significantly increased risks of STS from exposures to these compounds. Odds ratio (OR) 2.7 (95% confidence Interval (CI) 1.9, 4.7) and OR 3.3 (95% CI 1.8, 6.1) were obtained for phenoxyacetic acids and chlorophenols respectively [4]. However, multiple subsequent case–control and cohort studies in several countries have demonstrated conflicting results.

Since 1969, some of the herbicides including 2,4,5-trichlorophenoxyacetic acid (2,4,5-T) were banned in Europe and the United States (US) due to contamination by 2,3,7,8-tetrachlorodibenzo-p-dioxin (TCDD) [5]. The latest update from the International Agency for Research on Cancer (IARC) published in 1987 concluded that there was a limited evidence for carcinogenicity to humans from chlorophenoxy herbicides (Group 2B) and inadequate evidence for carcinogenicity to animals from 2,4-dichlorophenoxyacetic acid (2,4-D) and 2,4,5-T [5]. Another herbicide—Agent Orange, a mixture of 2.4.5-T and 2,4-D—was studied in Vietnam War veterans as it was widely used as a defoliant during the Vietnam War [6]. The US National Academies of Sciences, Engineering and Medicine linked exposure to Agent Orange to certain cancer precursors and cancers, including soft-tissue sarcoma, in its most recent report titled Veterans and Agent Orange: Update 11 (2018) [7].

STS is one of the few specific tumors linked to dioxins. TCDD, which is considered as the most potent dioxin, is classified as a group 1 carcinogen by the IARC. This is based on sufficient evidence in experimental animals and limited evidence in humans [8]. While the human epidemiological evidence showed an excess of all cancer mortality in four highly exposed industrial cohorts to dioxins [9–12], the association of exposure to dioxins and sarcomas was inconsistent.

Vinyl chloride monomer (VCM) is another compound studied in relation to angiosarcoma of the liver (ASL) and other soft-tissue sarcomas. The first reports on cases of ASL among workers occupationally exposed to VCM appeared in 1974 [13–16]. The IARC classifies VCM as a group 1 carcinogen, with sufficient evidence of carcinogenicity in humans [17].

Various occupational exposures have been studied to examine sarcoma incidence and mortality with inconsistent results. We aimed to include new epidemiological evidence in our systematic review and meta-analysis. The objective of this study is to explore the incidence and mortality of sarcomas in adult population with various occupational exposures.

## Methods

### Search strategy

We conducted a systematic literature search using the PubMed, Scopus, Embase, and Cochrane databases, as well as using backward citation tracking, to search for all reports of cohort or case–control studies that provided data on the risk of sarcoma in relation to occupational factors published up to May 2021. The literature review and meta-analysis was carried out and reported according to the Preferred Reporting Items for Systematic Reviews and Meta-Analysis (PRISMA) recommendations [18] (Supplementary table 8). The systematic review protocol and search strategy were registered online (PROSPERO May 7, 2020: CRD420201809).

The literature search was developed by two medical research librarians (GA and DG). In order to identify relevant studies, we used terms that combined various occupational exposure types with outcomes of interest—incidence and mortality of sarcomas. The full search strategy is available in supplementary table 1.

### Study selection

After removal of duplicates, two authors (DE and AV) independently identified studies eligible for inclusion based on an initial screen of reference titles and abstracts. The inclusion and exclusion criteria for this review are provided in Table 1. Studies found to be relevant to the topic of interest were shortlisted. The shortlisted publications were retrieved for an independent full-text article review and were assessed for the eligibility criteria by the same authors. The articles that met all inclusion criteria were included for final systematic review. Any discrepancies during the search process were resolved by the lead investigator (AG). We used EndNote for management of references.

**Table 1:** Inclusion and exclusion criteria for screening articles

Domain	Study inclusion criteria	Exclusion criteria
Study type/design	Prospective or retrospective cohort and case–control studies	Reviews, case reports, meeting abstracts, notes, letters, comments, and editorials
Study population	Adult population (older than 18 years) with occupational exposure	
Comparator	No occupational exposure	
Exposure type	Various occupations, chemicals and compounds, including herbicides, pesticides, Agent Orange, dioxins, vinyl chloride monomers and others	Radiation exposure, thorotrast exposure, and non-occupational exposures
Duration of exposure	No restriction	
Outcome	Sarcoma incidence or mortality	Cancers other than sarcoma
Subtype of sarcoma	Any sarcoma subtype	Kaposi sarcoma
Time of publication	No restriction	
Publication type	Full-text published article	
Language	English, French, Russian	Studies published in a language other than English, French or Russian

### Data extraction and quality assessment

Two authors (DE and AV) independently extracted data using a standardized form. The publications that met the inclusion criteria were abstracted onto tables, separately for cohort and case–control studies. The data included author, year of publication, country, time period covered, exposure duration, exposure type, description of study and reference groups, outcome of interest, and risk estimates for relevant outcomes. The effect estimates that were abstracted were odds ratio (OR), risk ratio (RR), standardized incidence ratio (SIR) or standardized mortality ratio (SMR) estimates, and their 95% confidence intervals. Studies that did not report the later were excluded, and no crude numbers were abstracted to compute effect estimates. For cohort studies, risk estimates took the form of standardized mortality or risk ratios. For case–control studies, risk estimates were presented as odds ratios. The quality of case–control and cohort studies was assessed using the Newcastle–Ottawa Scale [19]. Cofounders in the studies were included as part of our risk of bias assessment. When cofounders were not adjusted for in the study the outcome risk of bias was judged as high.

### Data synthesis and statistical analysis

We used Microsoft Excel for data abstraction. The primary meta-analyses were conducted to assess the association between various occupational exposures and sarcoma incidence and mortality. Pooled effect estimates of ORs, RRs, SIRs or SMRs, and 95% CI were calculated using a random-effects model. Forest plots were generated, displaying the individual study effects and the pooled estimate effect. Heterogeneity between individual studies was assessed by the chi-square test (by the *Q* statistic) and *I*^2^ statistic, which represents the amount of variability in the meta-analysis attributed to study heterogeneity [20]. An *I*^2^ of < 25% was considered as low-level heterogeneity and 25 to 50% as moderate level. *P* ≤ 0.10 and/or *I*^2^ > 50% indicated significant heterogeneity [21]. All analyses were conducted in SAS v9.4 (Cary, NC).

## Results

### Study characteristics

**Fig. 1:**
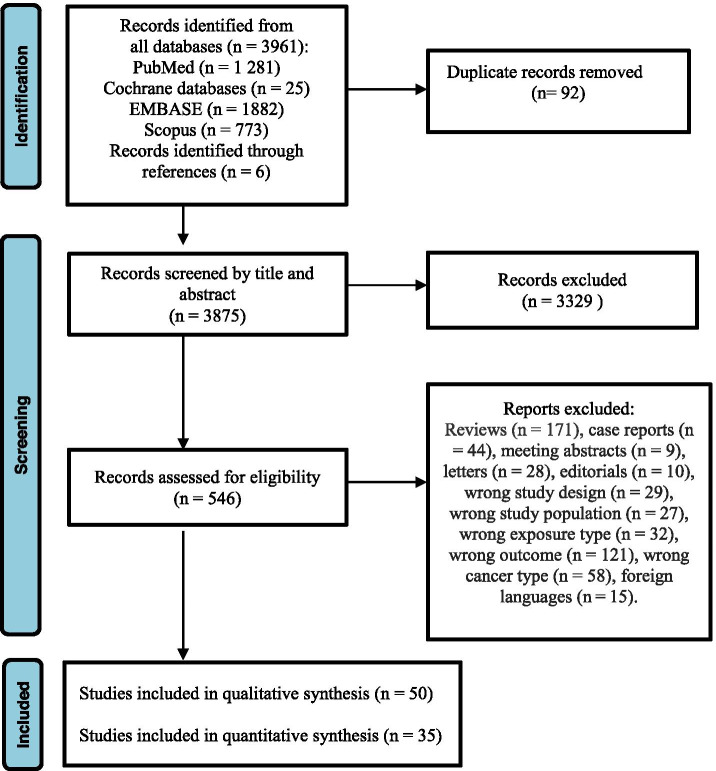
PRISMA flow diagram

Our literature search identified an initial 3961 publications of which 92 duplicate articles were removed. We excluded 3329 articles based on title and abstract screening. We retrieved the 546 remaining articles for detailed full article screening for specific inclusion and exclusion criteria as well as for overlapping data. For duplicate reports, we selected the studies with larger sample size and longer follow-up time. A total of 50 studies met full eligibility criteria to be included in qualitative data synthesis and 35 studies were included in meta-analysis (Fig. 1). Fifteen studies were not included in the meta-analysis because the described exposures or occupations were not studied in any other study.

### Exposure to phenoxy herbicides and chlorophenols

We included 17 case–control [22–38] and 10 cohort studies [38–47] that provided data on occupational exposures to phenoxy herbicides and chlorophenols. We presented the characteristics of individual studies in Tables 2 and 3 and the findings of these studies in supplementary tables 2 and 3.

**Table 2:** Characteristics of case–control studies of phenoxy herbicides and chlorophenols

Author/year	Region/country	Time period	Age/gender	Cases	Controls
Hardell et al., 1979 [22]	Northern Sweden	1970–1977	Male 26–80	52 histologically confirmed soft tissue sarcoma (STS) patients admitted to the department of oncology	Age, sex, and place of residence matched 208 controls from the National Population Registry
Eriksson et al., 1981 [23]	Southern Sweden	1974–1978	Male	110 histologically confirmed STS cases reported to the cancer registry of the National Social Welfare Board	By municipality and age matched 220 controls extracted from the National Population Registry
Greenwald et al., 1984 [24]	US	1962–1980	Male	281 men with Vietnam service and military service experiences who were diagnosed with STS	281 controls derived from driver’s license files and matched on 5-year period of birth and ZIP code of residence
Smith et al., 1984 [25]	New Zealand	1976–1980	Male	82 subjects with histologically confirmed STS, reported to the National Cancer Registry from public hospitals	92 controls with other types of cancer matched on age and had the same year of registration
Hoar et al., 1986 [26]	Kansas, US	1976–1982	Male > 21	All newly diagnosed 133 STS cases through the University of Kansas Cancer Data Service	948 controls from general population of Kansas, matched on age and vital status
Kang et al., 1986 [27]	US	1969–1983	Male	234 Vietnam-era veteran patients who served in the US military between 1964 and 1975, treated in one of the 172 Veterans Affairs hospitals with a diagnosis of STS	13,496 Veterans Affairs patients who were sampled from the same Vietnam-era veteran patient population from which the cases were drawn
Kang et al., 1987 [28]	US	1975–1980	Male	217 of STS sarcoma cases selected form the Armed forced Institute of pathology, men who were diagnosed STS between 1975 and 1980	599 controls from the Vietnam service population
Vineis et al., 1987 [29]	Northern Italy	1981–1983	Female/male > 20	68 persons (31 women) histologically revised STS cases diagnosed between 1981 and 1983, majority of cases were employed in rice weeding for > 10 years	158 randomly selected population referents (73 women)
Woods et al., 1987 [30]	Western Washington, US	1981–1984	Male 20–79	128 men with STS identified through a population-based tumor registry that covered 13 counties	694 randomly selected live controls without cancer
Wingren et al., 1990 [31]	Southeast Sweden	1975–1982	Male 25–80	96 STS cases	450 randomly selected population referents, including 200 individuals with a cancer diagnosis other than sarcoma
Eriksson et al., 1990 [32]	Seven counties of Central Sweden	1978–1986	Male 25–80	218 histologically confirmed STS cases	212 controls, matched by age, gender, and county of residence
Franceschi et al., 1992 [33]	Friuli-Venezia Giulia region, Northeast Italy	1985–1991	Female/male 16–79	93 STS cases (53 males and 40 females), admitted in-patient or outpatient clinics of the Avino cancer center or to the general hospitals of the study area	721 controls: 371 males and 350 females admitted to aforementioned hospitals for a wide spectrum of disease, malignant disorders were excluded
Smith et al., 1992 [34]	Victoria, Australia	1976–1987	Male > 30	30 males with STS treated at any of six major Melbourne hospitals and who were still alive at the time of selection for the study	For each case Cancer Registry staff randomly selected one control with another type of cancer
Kogevinas et al., 1995 [35]	11 countries	1991	Female/male	21,183 workers from 24 cohorts. 13,898 workers were assessed as exposed to any phenoxy herbicide or chlorophenols, 3951 as unexposed, and 541 as of unknown exposure on the basis of job history. 11 STS cases were identified	5 controls per case, matched on age, sex, and country of residence at the time of employment
Hoppin et al., 1998 [36]	US	1984–1988	Male 32–60	251 STS cases, from eight population-based cancer registries: Connecticut; Kansas; Iowa; Miami, Florida; Detroit, Michigan; San Francisco, California; Seattle, Washington; and Atlanta, Georgia, men born in 1929–1953 to include the age group eligible for service in Vietnam	1908 male living controls, enabling more complete job history
Pahwa et al., 2011 [37]	Six provinces of Canada	1991–1994	Male ≥ 19	357 cases with first diagnosis of STS in 1991–1994, ascertained from provincial cancer registries, except in Quebec, where hospital ascertainment was used	1506 randomly selected, age matched, free of cancers of interest living controls, resident in the same province as cases
Coggon et al., 2015 [38]	UK	Up to 2012	Male	15 men with STS employed at five factories in England, which had manufactured phenoxy herbicides, or in a contract spraying business	Age-matched 150 controls

**Table 3:** Characteristics of cohort studies of phenoxy herbicides and chlorophenols

Author/year	Country	Time period	Age/gender	Study cohort	Reference cohort
Wiklund et al., 1986 [39]	Sweden	1961–1979	Male	354,620 workers employed in agriculture or forestry according to a national census in 1960, born in 1891–1940	1,725,845 men employed in other industries, born in the same time period
Wiklund, et.al., 1988 [40]	Sweden	From the date of license to 1984		20,245 Swedish pesticide applicators, observed by means of the Swedish Cancer Register	General Swedish population
Saracci et al., 1991 [41]	International study of 36 cohorts from 10 countries	1951–1987	Female /male	18,390 workers comprise 16,863 males, 1527 females, and 1 of unknown sex, ever-employed in production or spraying of phenoxy herbicides, including those contaminated by 2,3,7,8-tetrachlorodibenzo-p-Dioxin (TCDD)	General population
Sathiakumar et al., 1992 [42]	Alabama, US	1951–1987	Male	4323 men employed at a plant in Alabama that manufactures agricultural, including insecticides, herbicides, fungicides, micronutrients	US and AL general population mortality rates
Wiklund et al., 1994 [43]	Sweden	1971–1987	Female	50,682 women with occupations in agriculture, at least 20 h a week, according to the Swedish 1970 census	General female population
Gambini et al., 1997 [44]	Northern Italy	1957–1992	Male	1487 male rice growers	National death rate
Lynge, 1995 [45]	Denmark	1947–1993	Female/male	2119 workers from Denmark who were potentially exposed to phenoxy herbicides	General population of Denmark
Fleming et al., 1999 [46]	Florida, US	1975–1993	Female/male	33,658 (10% female) licensed pesticide applicators	General population of Florida
Alavanja et al., 2005 [50]	Iowa and North Carolina, US	Through 2006	Female/male	52,394 licensed private pesticide applicators in Iowa and North Carolina, 32,346 spouses of these private applicators, and 4916 licensed commercial applicators, recruited in 1993–1997	Total population of Iowa
Coggon et al., 2015 [38]	England and Wales	Though 2012	Male	8036 men employed at five factories, which had manufactured phenoxy herbicides, or in spraying business	General population of England and Wales

The earlier study conducted in 1979 by Hardell et al. in Northern Sweden demonstrated a six-fold increased risk of STS with exposure to phenoxy herbicides [22]. A southern Swedish case-referent study [23] with a large number of histologically proven cases (*n* = 110) confirmed the earlier findings of an increase of the same magnitude in the risk for STS after exposure to phenoxy herbicides and chlorophenols. The third Swedish study [31] also concluded that occupations which may imply exposure to phenoxy herbicides and chlorophenols, such as gardeners and railroad and wood workers, had an increased risk of STS.

Studies from New Zealand [25] and Australia [34] failed to confirm the Swedish findings. This study in New Zealand was conducted on a similar scale as the Swedish studies with regard to the number of STS cases. However, the study design differed from the Swedish studies by the control group consisting of other cancers. In a large population-based study conducted in six Canadian provinces with diverse agricultural practices Pahwa et al. also came to conclusion that STS was not associated with exposure to phenoxy herbicides [37]. The study limitations were low response rate and potential for exposure recall-bias. In a population-based study from Northern Italy, Vineis et al. found an increased risk of STS in female rice weeders, but not in male [48]. In another study from Italy, which was hospital-based, Franceschi et al. found no significant association of STS risk with exposure to herbicides [33]. Both Italian studies were limited by low statistical power and possible recall-bias. Two population-based studies undertaken in Kansas (Hoar et al., 1986) and Western Washington (Woods et al., 1987), states with high use of herbicides, found no association between agricultural herbicide use and the occurrence of STS [26, 30].

In contrast, the results of the largest US population-based study (Hoppin et al., 1998) that included 295 male STS cases from eight population-based cancer registries supported the hypothesis of an association of sarcoma with exposure of chlorophenols in cutting oils and wood preservation [36]. The findings of two-nested case controlled studies (Kogevinas et al., 1995) within a large international cohort including 21,183 workers, with substantial exposure to herbicides in production workers and sprayers also reported excess risk of STS [35].

As for Agent Orange, in a hospital-based study, Kang et al. concluded that there was no significant association of STS and previous military service in Vietnam [27]. However, the authors noted that the absence of positive association might be due to insufficient observation time. Another study from the same group of researchers compared a total of 217 STS cases to 599 controls for Vietnam Service [28]. The study revealed that Vietnam veterans in general did not have an increased risk of STS when compared to those men who had never been in Vietnam. However, an increased risk of STS was observed in the subgroups of veterans, such as combat veterans, combat veterans with MOSC (military occupational specialty category), and those who served within military region III, the area where Agent Orange spray was reported to be excessive.

In a cohort study of workers in manufacture of phenoxy herbicides in Denmark, findings of Lynge et al. supported the earlier Swedish observations of an excess risk of STS following exposure to herbicides [49]. In contrast, in the Swedish study with 15% estimated risk of exposure to phenoxy herbicides in agricultural or forestry workers with large numbers of STS cases (*n* = 331), Wiklund et al. reported no increased risk of STS [39]. The findings from two other cohort studies of Swedish licensed pesticide applicators [40] and Swedish female farmers [43], presented by the same group of authors, were consistent with the hypothesis that exposure to these compounds does not increase risk of STS. These studies were limited by lack of information on exact exposure to herbicides and consisted of cohorts with assumed occupational exposure to phenoxy acids. Data from ongoing large prospective cohort study of farmers and pesticide applicators in North Carolina and Iowa called the Agricultural Health Study also did not show increased risk of STS [50]. Similarly, Fleming et al. found no cases of STS in a large retrospective cohort study of 33,658 pesticide applicators from Florida [46].

Increased mortality from STS was on the border of statistical significance among workers at a plant in Alabama manufacturing agricultural and other chemicals, based on small numbers (n = 3) [42]. Similarly, in the study of rice growers in Northern Italy Gambini et al. were unable to come to any firm conclusion as there was only one case of death from STS with SMR as high as 1808.5 due to the very low expectation in this category [44].

In a large international cohort of chemical workers from ten courtiers in manufacturing agricultural and other chemicals, set up by the IARC, Saracci et al. found a six-fold excess of STS in the cohort as a whole and a nine-fold excess among sprayers 10–19 years from first exposure [41].

### Exposure to dioxins

We included 4 cohort studies that provided data on occupational exposures to dioxins [51–54]. The studies evaluating non-occupational exposures and industrial accidents were not included. We presented the characteristics of the individual studies in Table 4 and the findings of these studies in supplementary table 4.

**Table 4:** Characteristics of cohort studies for occupational exposures to dioxins

Author/ Year	Region/country	Exposure period	Follow-up period	Age/gender	Study cohort	Reference cohort
Kogevinas et al., 1997 [51]	12 countries	1939–1992	1939–1992	Female/male	21,863 workers in 36 cohorts with substantial exposure to dioxins	National mortality rates
Steenland et al., 1999 [52]	United States (US)	1942–1984	Trough 1993	Female/male	5132 workers at 12 US plants producing 2,3,7,8-tetrachlorodibenzo-p-dioxin (TCDD) contaminated chemicals, including Agent Orange	US population
Bodner et al., 2003 [53]	US	1940–1984	1940–1995	Male	Dow Chemical Company cohort of 2187 male dioxin exposed workers	US population
Collins et al., 2009 [54]	Michigan, US	1942–1982	1942–2003	Female/male	1615 workers working > 1 days in a department with potential TCDD exposure	US population

The largest study was a historical cohort of 21,863 male and female workers from 12 countries coordinated by the IARC and followed for 70 years [51]. In this study, Kogevinas et al. observed twofold excess mortality for STS among workers exposed to herbicides contaminated with dioxins, subjects with a long duration of exposure, and persons first employed before 1965. The validity of this study may have been influenced by a possibility of exposure misclassification and inaccurate STS diagnosis on death certificates. Steenland et al. found excess mortality in the highest exposed chemical workers at 12 US plants [52]. Bodner et al. reported a greater than expected rate for STS in another US cohort of chemical production workers exposed to substantial levels of dioxin [53]. Collins et al. also observed an increased mortality rate from STS with dioxin exposure in the latest update from the largest single plant cohort study of workers at the Dow Chemical Company in Michigan with a follow-up period of 62 years [54]. Limitations of this study were the potential for misclassification of sarcoma diagnosis and the small number of sarcoma cases.

### Exposure to vinyl chloride monomers

We included 5 cohort studies [55–59] observing the relationship to mortality from ASL. Three of these studies also evaluated the association between VCM and other STS. Two multicenter epidemiologic studies combined populations of exposed workers included in previous investigations and extended the follow-up. We presented the characteristics of the individual studies in Table 5 and the results of these studies in supplementary table 5.

**Table 5:** Characteristics of cohort studies of exposures to vinyl chloride monomer (VCM)

Author/year	Country	Years of employment	Years of follow-up	Study cohort	Reference cohort
Smulevich et al., 1988 [55]	Former Soviet Union	1939–1977	1939–1977	3232 workers (2195 men, 1037 women) with > 1-month employment of the vinyl chloride (VC) and polyvinyl chloride (PV) chemical plant	General population of the city where the plant was located
Ward et al., 2001 [56]	Italy, Norway, Sweden, UK	1940–1989	1955–1997	12,706 workers from 19 plants of VC, VCM and PVC production, with ≥ 1 year employment	Age and calendar period specific national incidence or mortality rates from the World Health Organization
Wong et al., 2002 [57]	Taiwan	1950–1992	1985–1997	3293 workers from 6 polyvinyl chloride polymerization factories employed ≥ 1	General Taiwanese male population
Mundt et al., 2017 [58]	US and Canada	1942–1972	1942–2013	9951 VC or PVC resin manufacture workers at 35 factories in the US and Canada employed for ≥ 1 year	General population
Fedeli et al., 2019 [59]	Italy	1956–1985	1973–2017	1658 workers employed in a VCM production and polymerization facility in Porto Marghera (northeastern Italy) 1658 workers employed in a VCM production and polymerization facility in Porto Marghera (northeastern Italy) 1658 male workers employed in VCM production and polymerization facility	General population

The European multicenter study (Ward et al., 2001) [56] was an extended follow-up of a cohort organized by Simonato et al. [60] and included the population previously studied in the UK [61, 62], Sweden [63], and Norway [64]. The results were generally consistent with the original findings in which the excess from liver cancer was related to time since first exposure, duration of employment and estimated quantitative exposures. A strong relation was observed between cumulative exposure to VCM and occurrence of liver cancer. The relationship was even more evident when only ASL was analyzed.

Mundt et al. aimed to evaluate exposure–response relationships for mortality from ASL and hepatocellular carcinoma (HCC) in the North American cohort mortality data for men engaged in the manufacture of VC or PVC resin at any of 35 factories in the US [58]. The association between VCM and ASL reported in this cohort 44 years ago persisted in the updated study and was strongest among most highly exposed workers. These findings were consistent with those reported in the European study (Ward et al., 2001). The median latency for ASL deaths was 36 years.

Collins et al. reported 15 cases of ASL in the most recent update of the Dow chemical company diseases registry for ASL [65]. Thirteen ASL cases were at a single plant with high VCM exposure before 1974, which could indicate that exposures were higher at this location than the other locations. Fedeli et al. reported 9 deaths from ASL in a cohort of 1658 workers involved in VCM production and polymerization in northeastern Italy [59]. Latency among ASL cases was 32 years and the risk continued to increase through the highest levels of cumulative exposure. Wong et al. found increased mortality from HCC but no death caused by ASL in the retrospective cohort study from Taiwan [57]. This could be due to much lower levels of VCM exposure in Taiwanese workers compared to North American and Western European workers.

### Other occupation exposures

We included 6 cohort studies [66–71] and 12 case–control studies [31, 33, 37, 72–79] that provided data on various other occupational exposures and sarcoma incidence and mortality. We presented the characteristics of the individual studies in Tables 6 and 7and the results of these studies in supplementary tables 6 and 7.

**Table 6:** Characteristics of cohort studies of other occupational exposures

Author/year	Country	Employment	Gender/age	Study cohort	Reference cohort
Polednak et al., 1978 [66]	US	1915–1929	Female	634 employees, who worked in the US radium dial-painting industry	US white female population
Teta et al., 1988 [67]	US	1946–1981	Male	846 employees of a research, engineering, and metal fabrication facility	US white male population and the white male population of Niagara and Erie counties in New York state
Wiggs et al., 1994 [68]	US	1943–1977	Male	15,727 white employees	US white male population
Rix et al., 1998 [69]	Denmark	1943–1993	Female/male	14,362 Danish paper mill workers employed at any time between 1943 and 1990	National cancer rate was used to calculate expected cancer cases
Rix, Villadsen, and Lynge, 1997 [70]	Denmark	1955–1993	Female/male	2238 workers employed in 1955–1990 at two Danish sulfite mills	National cancer rate was used to calculate expected cancer cases
Koshurnikova et al., 2000 [71]	US/Russia	1948–1958	Female/male	11,000 employees of ‘‘Mayak’’ nuclear reactor and plant, plutonium production facility	General US and Russian populations

**Table 7:** Characteristics of case–control studies of other occupational exposures

Author/year	Region/country	Timeperiod	Age/gender	Cases	Controls
Balarajan et al., 1984 [80]	England and Wales	1968–1976	Male	1961 persons diagnosed with malignant neoplasm of connective tissue and other soft tissue	1961 men with any other cancer from the National Cancer Register with a valid occupational code randomly matched for age and region of residence
Hoar Zahm et al., 1988 [72]	Kansas, US	1976–1982	Male > 21	All newly diagnosed 133 soft tissue sarcoma (STS) cases among Kansas residents, identified through the University of Kansas Cancer Data Service	948 male from general population of Kansas
Pearce et al., 1988 [73]	New Zealand	1980–1984	Male > 20	A series of case-referent studies based on the New Zealand Cancer Register and involving 19,904 male cancer patients. Total number of STS case was 171, with occupation recorded—142	For each site, the registrants for other sites formed the reference group
Zahm et al., 1989 [74]	Kansas, US	1976–1982	Male > 21	All newly diagnosed 133 STS cases among Kansas residents, identified through the University of Kansas Cancer Data Service	948 white men from general population of Kansas matched by age and vital status
Wingren et al., 1990 [31]	Sweden	1975–1982	Male	96 STS cases	450 randomly selected population referents and 200 cancer referents
Franceschi et al., 1992 [33]	Northern Italy	1985–1991	Male/female	93 STS cases (53 male and 40 female)	Control group consisted of 721 patients (371 men and 350 women) admitted to the aforementioned hospitals for a wide spectrum of diseases. Excluded patients with malignancies
Hoppin et al., 1999 [75]	US	1984–1988	Male 30–60	251 living sarcoma cases from eight population-based cancer registries, born between 1929 and 1953 to include the age group eligible for service in Vietnam	1908 living controls
Briggs et al., 2003 [76]	US	1984–1988	Male	335 confirmed cases of STS born 1929–1953, diagnosed with cancer in 1984–1988	2290 selected, 1910 completed interviews
Pahwa et al., 2003 [77]	Canada	1971–1991	Male	365 living males with STS	1506 controls matched by age, province, and sex, selected from population-based sources within each province
Merletti et al., 2006 [78]	9 Western European countries	1995–1997	Female/male	96 cases of STS interviewed born in 1925–1961, diagnosed in 1995–1997	2632 randomly selected population controls
Hossain et al., 2007 [79]	Canada	1991–1994	Male > 18	357 men with STS, diagnosed in 1991–1994, identified from provincial cancer registry	1474 controls, matched by age and resident in the same provinces
Pahwa et al., 2011 [37]	Canada	1991–1994	Male ≥ 19	357 cases with first diagnosis of STS in 1991–1994, ascertained from provincial cancer registries, except in Quebec, where hospital ascertainment was used	1506 controls matched by age, province, and sex, selected from population-based sources within each province

Zahm et al. demonstrated an association between woodworking occupations and increasing risk of STS in a population-based case–control study in Kansas[74]. Hoar et al. found increased risk of STS with exposure to insecticides used on animals but not on crops [72]. Similarly, Pahwa et al. observed a statistically significant increased risk of STS with exposure to insecticides—aldrin and diazinon in Canadian population-based case–control study [77]. STS risk was higher among the farmers with longer duration of exposure, farmers who themselves mixed or applied insecticides to animals and failed to use any protective equipment.

In a large historical cohort of Danish paper mill workers, Rix et al. found an increased risk of STS female workers with a high risk among paper sorters employed in manual sorting and packing [69]. Another cohort form the same authors found an excess risk of STS in Danish sulfite pulp workers [70].

In a large case–control study from six Canadian provinces, Hossain et al. found an increased risk for STS associated with an exposure to radium, longest-held job as a machinist, short-term job as chicken farm worker, pulp and paper industry worker, and apartment complex worker [79].

A large population-based study from the US reported by Hoppin et al. demonstrated excess risk of different sarcoma subtypes from various occupational exposures [75]. In this study, self-reported herbicide use and exposure to chlorophenols and cutting oil was associated with malignant fibrohistiocytic sarcoma and leiomyosarcoma, wood-related exposures with leiomyosarcoma, and meatpacking with dermatofibrosarcoma protuberans. In another large population-based case–control study from the US, Briggs et al. showed that exposure to wood dust was associated with increased risk of STS in African American men but not in white men [76]. Race-specific occupational risk factors evident only among African American men may represent racial disparities in levels of exposures to carcinogens.

In a case–control study from Sweden, Wingren et al. demonstrated that gardeners, railroad workers, unspecified chemical workers, workers in contact with wood, and construction workers with exposure to asbestos and pressure impregnating agents had an increased risk of STS [31]. In a case–control study from Northern Italy, Franceschi et al. came to the conclusion that workers with exposure to chemical agents, benzene, or other solvents had higher risk of developing STS [33]. Excess mortality in bone and soft tissue sarcomas was detected in a large cohort of US and Russian “Mayak” nuclear facility workers exposed to plutonium [71]. In a series of case-referent studies from the New Zealand, Pearce et al. observed that the risk for STS was elevated in meat workers [73]. In the study conducted in England and Wales, Balarajan et al. found no increased risk of STS among farmers and allied workers. However, when each occupational subgroup was analyzed separately, the excess risk found was limited to farmers, farm managers, and market gardeners [80]. In the largest international case–control study, conducted in nine European countries, Merletti et al. found increased risk of bone sarcoma in woodworkers (particularly carpenters), blacksmiths, toolmakers and machine-tool operators, workers employed in manufacture of equipment and machinery industry, construction workers, and workers who ever used pesticide [78].

### Results of meta-analysis

#### Exposure to phenoxy herbicides and chlorophenols

**Fig. 2:**
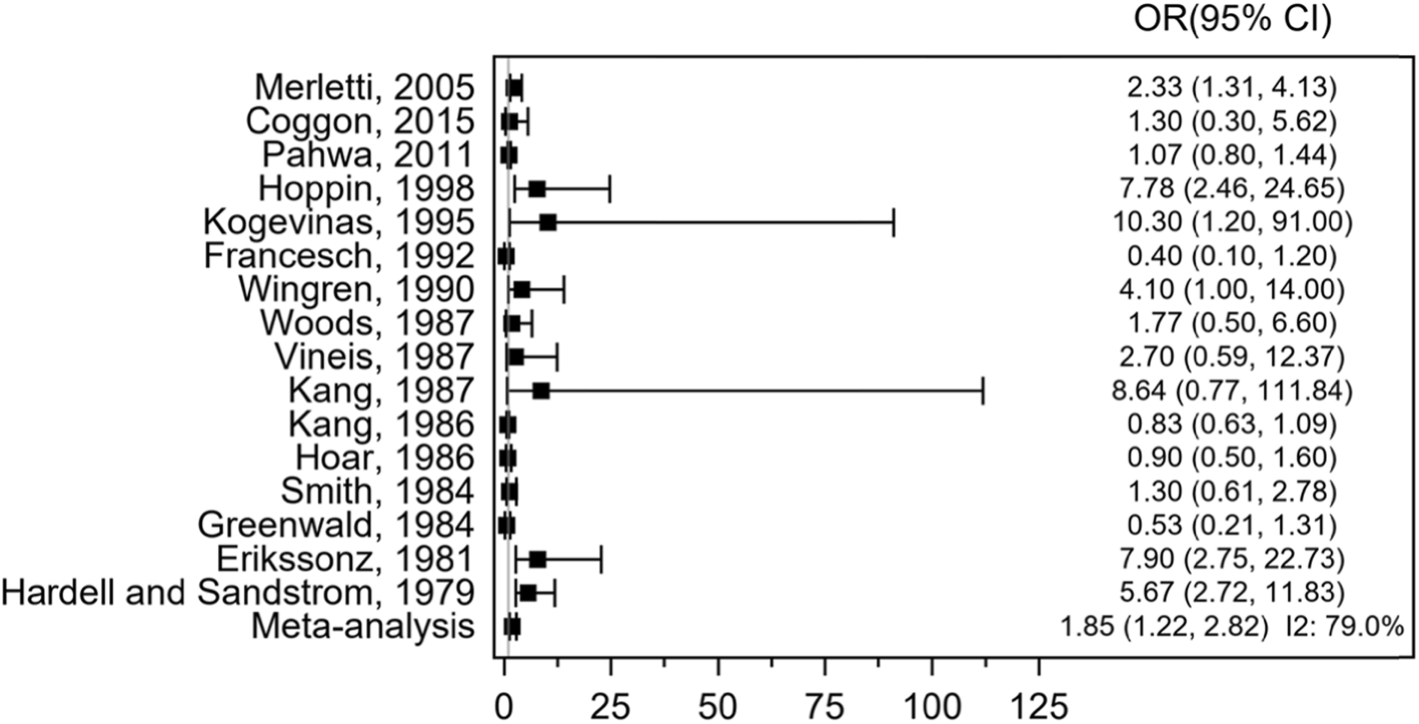
Forest plot of odds ratio (OR) for exposure to phenoxy herbicides and chlorophenols and sarcoma incidence

We conducted a meta-analysis of 16 case–control studies, involving 2254 sarcoma cases and 24,148 con1trols (Fig. 2). The pooled OR was 1.85 (95% CI 1.22, 2.82), *P* = 0.008, indicating significant positive association between exposure to phenoxy herbicides and chlorophenols and incidence of sarcoma. There was significant heterogeneity across the individual studies *I*^2^ = 79.0%, *P* ≤ 0.001.

**Fig. 3:**
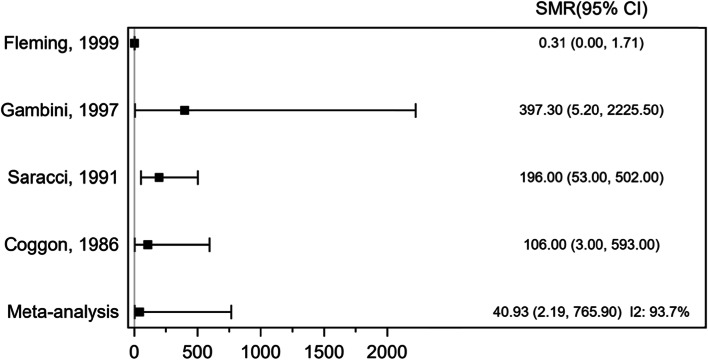
Forest plot of standardized mortality ratio (SMR) for exposure to phenoxy herbicides and chlorophenols and sarcoma mortality (cohort studies)

Four cohort studies involving 11 sarcoma cases and 59,289 participants assessed the association between exposure to phenoxy herbicides and chlorophenols and sarcoma mortality (Fig. 3). The pooled SMR was 40.93 (95% CI 2.19, 765.90), *P* = 0.013, indicating a statistically significant positive association. However, there was significant heterogeneity across the individual studies *I*^2^ = 93.7%, *P* ≤ 0.001.

**Fig. 4:**
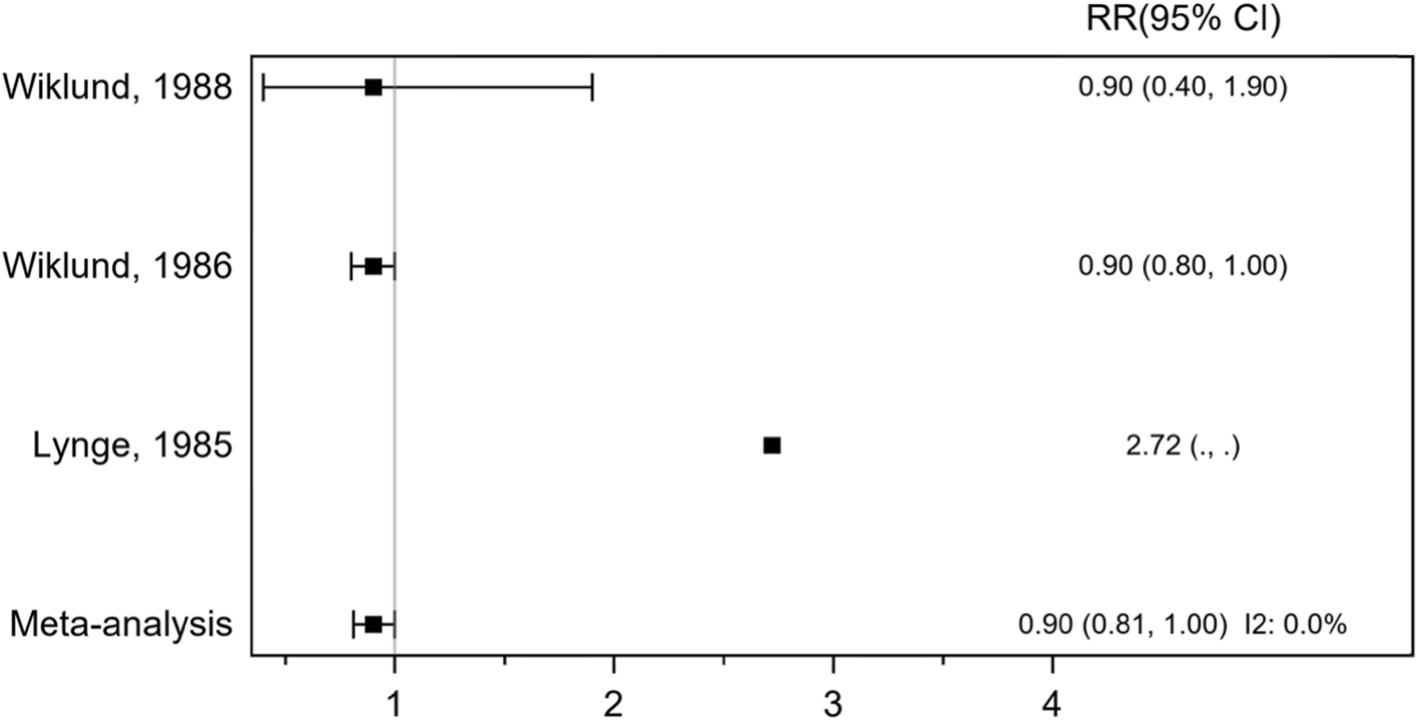
Forest plot of risk ratio (RR) for exposure to phenoxy herbicides and chlorophenols and sarcoma incidence (cohort studies)

Based on meta-analysis of 3 cohort studies, involving 379,864 participants and 343 sarcoma cases, assessing exposure to phenoxy herbicides and chlorophenols and sarcoma incidence, the pooled RR was 0.90 (95% CI 0.81, 1.00), *P* = 0.0489, indicating no association (Fig. 4). There was no evidence of heterogeneity across the included studies, *I*^2^ 0.0%, *P* = 1.00. Two studies included in this meta-analysis, Wiklund et al. [39]and Wiklund et al. [40], that showed no association between exposure to phenoxy herbicides and chlorophenols and STS were much larger in size and tended to drive the results of meta-analysis. One explanation for the lack of any excess of sarcoma in these studies might be that both studies were register-based studies consisted of cohorts of agricultural and forestry workers and pesticide applicators with assumed occupational exposures to phenoxy acids and lacked individualized exposure data.

**Fig. 5:**
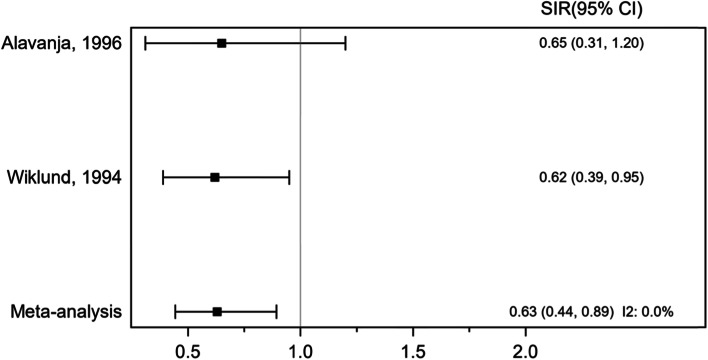
Forest plot of standardized incidence ratio (SIR) for exposure to phenoxy herbicides and chlorophenols and sarcoma incidence (cohort studies)

Based on 2 cohort studies, involving 103,078 participants and 31 cases of sarcoma, the pooled SIR was 0.63 (95%CI 0.44, 0.89), *P* = 0.010, indicating no association between exposure to phenoxy herbicides sarcoma incidence (Fig. 5). There was no evidence of heterogeneity across the included studies, *I*^2^ 0.0%, *P* ≤ 0.001.

#### Exposure to dioxins

**Fig. 6:**
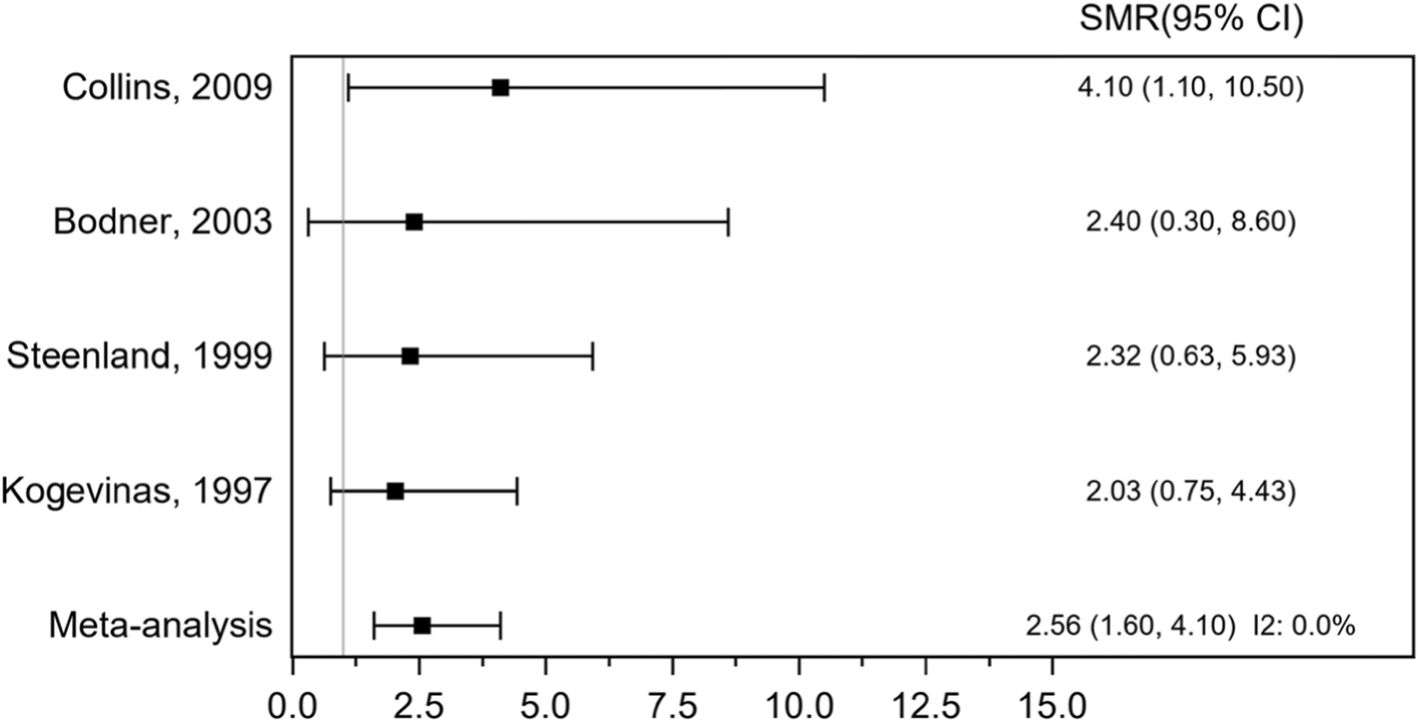
Forest plot of standardized mortality ratio (SMR) for occupational exposure to dioxins and soft tissue sarcoma (STS) mortality (cohort studies)

A meta-analysis of 4 cohort studies involving 30,797 participants evaluated the association between exposure to dioxins and STS mortality (Fig. 6). There were 14 deaths due to STS in these studies. The pooled SMR was 2.56 (95% CI 1.60, 4.10), *P* ≤ 0.001, indicating a statistically significant positive association between exposure to dioxins and STS mortality. There was no evidence of heterogeneity across the included studies, *I*^2^ 0.0%.

#### Exposure to vinyl chloride monomers

**Fig. 7:**
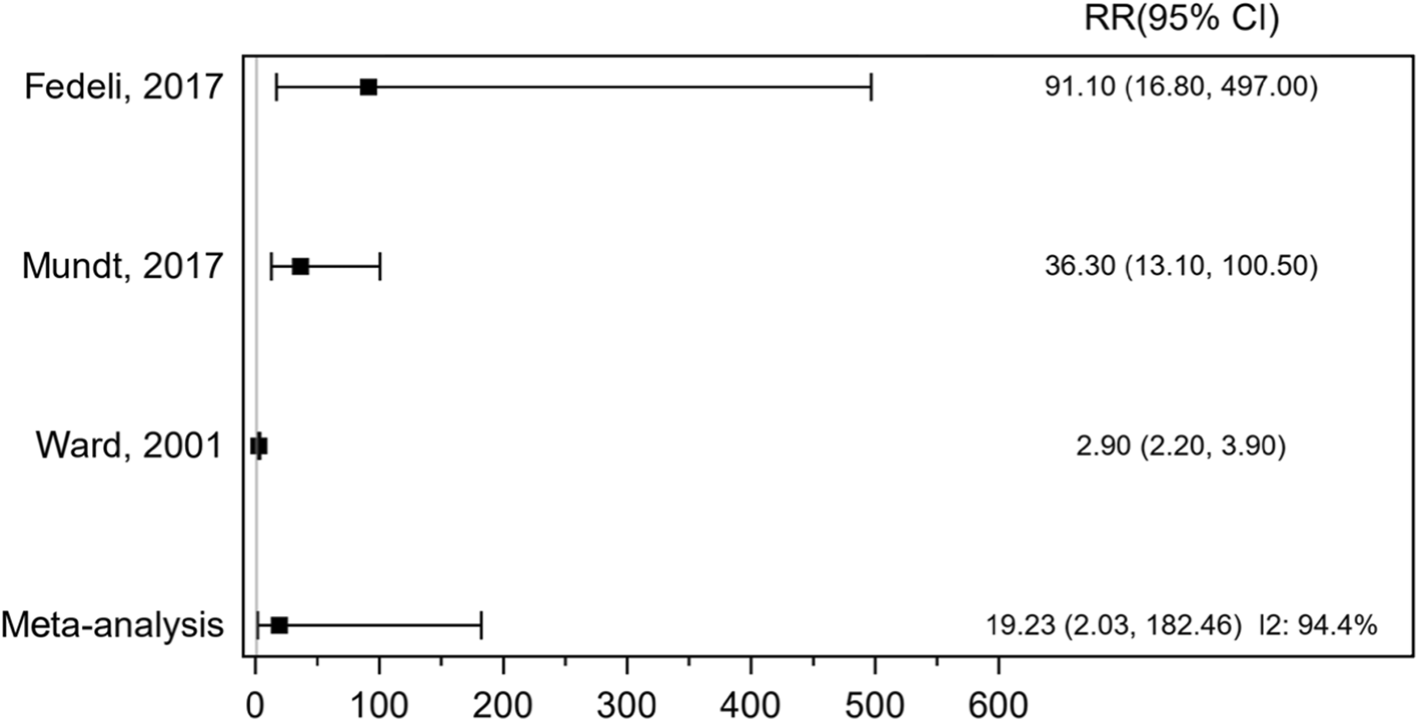
Forest plot of risk ratio (RR) for exposure to vinyl chloride monomer (VCM) and mortality of angiosarcoma of the liver (ASL)

We conducted a meta-analysis of 3 cohort studies, including 12,816 participants. There were 110 deaths from STS in these studies. The RR of ASL was increased in all studies included in meta-analysis. The pooled RR was 19.23 (95% CI 2.03, 182.46, *P* = 0.010), indicating a statistically significant positive association between exposure to VCM and ASL mortality (Fig. 7). There was significant heterogeneity across the individual studies *I*^2^ = 94.4%, *P* ≤ 0.001.

**Fig. 8:**
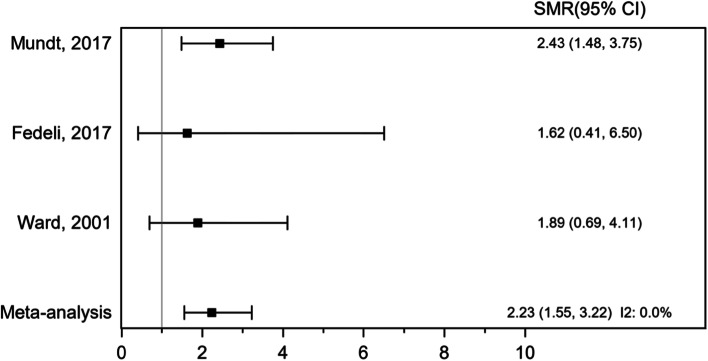
Forest plot of standardized mortality ration (SMR) for exposure to vinyl chloride monomer (VCM) and mortality of other soft tissue sarcomas

Excess death due to connective and soft tissue cancer was observed and was strongest amongst highly exposed workers in tree cohort studies including 12,816 participants (Fig. 8). There were 20 deaths due to connective and soft tissue cancer in these studies. The pooled SMR was 2.23 (95 CI 1.55, 3.22), *P* < 0.001, indicating statistically significant positive association between VCM exposure and STS. The study results were adequately similar, as *I*^2^ = 0.0%.

### Other occupational exposures

With regard to other occupational exposures, population-based studies reported increased sarcoma incidence with exposure to insecticides used on animals, benzene, radium, cutting oil, and wood dust, in female paper sorters, gardeners, railroad workers, farmers, farm managers, long-term jobs as a machinist, short-term jobs as chicken farm workers, temporary jobs at apartment complexes, pulp and paper industry workers, meatpacking and woodworking occupations, and sulfite mill and nuclear facility workers.

**Fig. 9:**
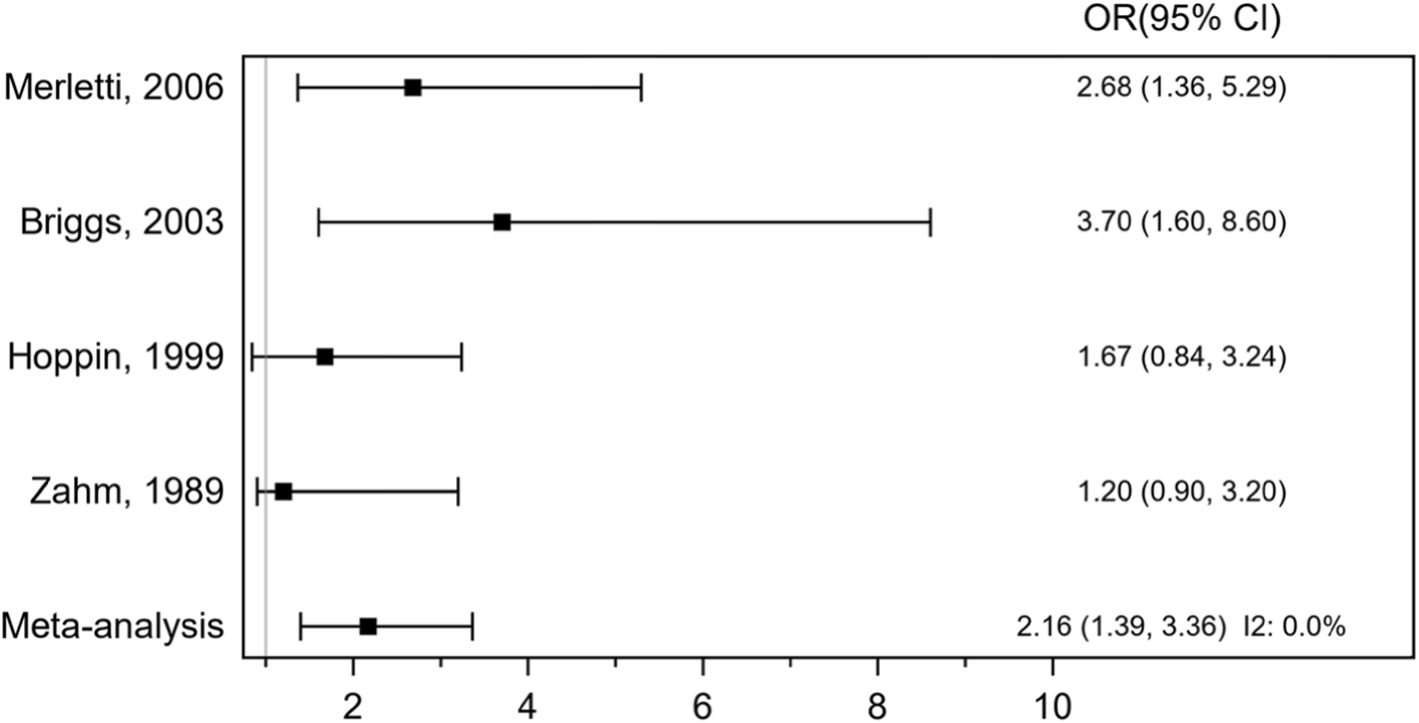
Forest plot of odds ratio (OR) for occupational exposure to woodworking and soft tissue sarcoma (STS) incidence

Meta-analysis of 4 case–control studies with 8593 participants assessed the association between STS incidence and occupational exposure to woodworking and wood dust (Fig. 9). The pooled OR was 2.16 (95% CI 1.39, 3.36), *P* < 0.001, indicating a statistically significant positive association. There was no significant heterogeneity across the studies, *I*^2^ = 0.0%.

## Discussion

Our systematic review and meta-analysis included a comprehensive review of the PubMed, Scopus, EMBASE, and Cochrane databases, allowing for a considerable amount of data collection. This is the largest study to date that evaluates the association between various occupational exposures and sarcoma incidence and mortality. Our study has multiple strengths. We have included studies from various geographical areas from several countries that add to the generalizability of our results. A broad mix of studies was analyzed, including different populations and a wide variety of occupational exposures. The total amount of participants was large and different study designs were included. Many studies confirmed their sarcoma diagnosis with review of histology, and long time for follow-up was preferred. The majority of included studies were rated between moderate and high quality of evidence (supplementary table 9).

However, there were several limitations noted both in case–control and cohort studies that contributed to the inconsistency of outcomes across the studies. We believe this inconsistency is most likely due to limitations faced with study design based on questionnaires that are prone to recall bias, among other biases including measurement bias from lack of accurate exposure levels among the different studies. These limitations were specifically noted in regard to exposures to phenoxy herbicides and chlorophenols. A number of studies have been limited by inadequate exposure information and difficulties in determining type, duration, and level of exposure in different jobs and different countries. The studies, in general, also lacked information on specific individual exposure level, on the proportion of exposed workers, or extent of exposure [39, 40, 43]. The occupational exposure level in the majority of studies was determined based on job title and exposure questionnaires [50]. In some studies, information on occupation was obtained from death certificates, enabling exposure misclassification bias [51]. Such misclassification of exposure may lead to either underestimation or overestimation of the relative risk, if not a systematic bias [81, 82]. In other studies, exposure information was collected from the study participants directly or from the next-of-kin, possibly allowing for recall bias [25, 34, 37]. Other reasons of inconsistency between the results of studies from different countries could be attributed to differences in type of herbicides used, level of dioxin contaminants, and climatic differences resulting in variability in agricultural practices [25, 30]. There is a possibility that in studies with positive results, the study population was exposed to higher cumulative levels of compounds or dioxin contaminants that were part of the commercial preparations [22]. Exposure risk evaluation for specific agents can be assessed only partially as workers often had simultaneous exposures to several different pesticides, engine fuels and exhausts, organic and inorganic dusts, UV radiation, heat, noise, vibration, mycotoxins, zoonotic viruses, and other biological agents [83, 84]. It would not be possible in the studies to identify a group of workers solely exposed to one specific agent.

Other possible reasons for a lack of consistency between the results may be differences between the study populations with respect to other uncontrolled confounding factors such as environmental conditions, lifestyle, and inherited factors. Main confounding factors considered in these studies were age and sex. Many studies excluded female workers due to small numbers and rarity of involvement in highly exposed occupations. Therefore, subgroup analysis based on gender was not performed due to population selection bias.

A number of cohort studies reported a limited statistical power and wide confidence interval, owing to the small number of sarcoma cases and low response rate [44, 54]. In some studies, the relative risk may have been underestimated because of the shorter follow-up period [27, 28]. The median latency period for the chemical induction of solid malignant tumors is generally considered to be in the range of 15–30 years [85, 86].

Another challenge across many studies was reliable and complete ascertainment of the cancers of main interest. Survival rates for sarcoma could be relatively high [87], meaning that cases will not necessarily be picked up from death certificates. Moreover, deaths from sarcoma have been often coded as cancers of the anatomical site at which they occur rather than as sarcoma specifically [88]. Sarcomas often present diagnostic difficulties that contribute to more misclassification bias [1]. The possibly of missing sarcoma cases due to incompleteness of cancer registration in many countries especially during early years of follow-up also could have influence on study outcomes. In several studies, death certificates were used to identify sarcoma cases, which add risk for misclassification of sarcoma and underestimation of sarcoma cases [51, 54].

With regard to VCM exposure, the included studies differed in design, size, length of follow-up, and covered time periods. Almost all studies concluded that the risk of malignant liver neoplasms, particularly ASL, was increased by exposure to VCM. However, the actual mechanisms of action of inducing ASL by VCM are unclear. The studies reported the highest incidence of ASL in workers of VCM production and polymerization. Some of the later observations report a dramatic decline in ASL cases from the late 1970s as a result of much stricter regulations implemented in North America and Western European countries to reduce VCM exposure. Most studies were unable to provide direct measurement of exposure to VCM and quantitative VCM exposure measure or analysis of liver cancer subtypes. The European multicenter study was the only study that provided a quantitative estimate of VCM exposure based on calendar-period-specific exposure, job, and plant. Some studies also noted difficulties in assessment of VCM as a cause of ASL or other STS because of lack of histological or other definitive clinical information to discriminate HCC from ASL. In most studies, the cause of death was obtained from death certificates that often incorrectly stated specific cause from liver cancer death and could have underestimated the number of cases of ASL and thus enabling misclassification bias. Only some studies provided SMR or SIR for ASL due to the rarity of the disease in the general population.

The results of our meta-analysis indicate that higher exposure to dioxins was associated with STS. However, the results were based on small number of STS due to rarity of sarcomas. Overall, included studies were good quality, with a large number of participants, long follow-up, and no significant heterogeneity across the included studies, which strengthen the validity of our findings.

Various other agricultural or non-agricultural exposures have been found to be associated with an increased risk of bone or soft-tissue sarcomas. However, due to limited number of studies, we were unable to conduct quantitative analysis for these occupations or exposures.

## Conclusion

Overall, our findings from the meta-analysis suggest a statistically significant positive association between higher exposure to dioxins and increased mortality from STS, between cumulative exposure to VCM and increased mortality from ASL and other sarcomas in organs other than the liver, and between woodworking occupation and exposure to wood dust and sarcoma incidence. Notwithstanding the high heterogeneity of the studies, workers exposed to phenoxy herbicides and chlorophenols may experience an increased incidence of sarcoma based on meta-analysis of case–control studies. Meta-analysis of cohort studies for exposure to chlorophenols and phenoxy herbicides, however, produced conflicting results.

Conducting new large case–control studies using better characterizations of exposure and extending follow-up of previously assembled cohort studies would be effective ways at reducing uncertainties and could provide more evidence on other risk factors reported in our study.

## Supplementary Information

**Additional file 1.** Supplementary tables.

## Data Availability

All data generated or analyzed during this study are included in this published article and its supplementary information files.

## Abbreviations

ASL: Angiosarcoma of the liver
CI: Confidence interval
HCC: Hepatocellular carcinoma
IARC: International Agency for Research on Cancer
OR: Odds ratio
RR: Risk ratio
STS: Soft-tissue sarcoma
SIR: Standardized incidence ratio
SMR: Standardized mortality ratio
VCM: Vinyl chloride monomer
2,4,5-T: 2,4,5-Trichlorophenoxyacetic acid
TCDD: 2,3,7,8-Tetrachlorodibenzo-p-dioxin
