# Supplementary material for: Association between occupational exposures and sarcoma incidence and mortality: systematic review and meta-analysis
Source: Syst Rev. 2021 Aug 13;10:231. doi: 10.1186/s13643-021-01769-4 (PMC8364027; doi:10.1186/s13643-021-01769-4)
Supplement: Supplementary file 1 — Additional file 1. Supplementary tables. [file 13643_2021_1769_MOESM1_ESM.docx]

Association Between Occupational Exposures and Sarcoma Incidence and Mortality: Systematic Review and Meta-analysis

D Edwards DO^1^, A Voronina DO^2^, K Attwood, PhD^3^ A Grand’Maison* MD^4^

^1^State University of New York at Buffalo, Department of Medicine

^2^NewYork-Presbyterian Queens, Department of Medicine

^3^Roswell Park Comprehensive Cancer Center, Department of Biostatistics

^4^Roswell Park Comprehensive Cancer Center, Department of Sarcoma Medical Oncology

**SUPPLEMENTARY MATERIAL**

**Supplementary table 1.** The full search strategy

| PubMed | ((((("Soft Tissue Neoplasms/epidemiology"[Mesh] OR "Soft Tissue Neoplasms/etiology"[Mesh])) OR ("Sarcoma/epidemiology"[Mesh] OR "Sarcoma/etiology"[Mesh] OR "Sarcoma/genetics"[Mesh] OR "Sarcoma/transmission"[Mesh])) AND (((("environmental exposure"[MeSH Terms] OR environmental exposure[Text Word])) OR (("Environmental Pollutants"[Pharmacological Action]) OR "Environmental Pollutants"[Mesh]) OR ("Occupational Exposure"[Mesh]) OR (("Carcinogens"[Mesh]) OR "Carcinogens"[Pharmacological Action]) OR ((((agrochemicals[MeSH Terms] OR Agricultural Workers Diseases[Mesh])) OR (2,4-Dichlorophenoxyacetic Acid[MESH] OR 2,4,5-Trichlorophenoxyacetic Acid OR Arsenicals[Mesh] OR arsenic[mesh] OR hydrocarbons, chlorinated[mesh] OR Benzofurans[mesh] OR Chlorophenols[mesh] OR Defoliants, chemical[MESH] OR defoliants, chemical[pharmacological action] OR "Dioxins and Dioxin-like Compounds"[mesh])) OR ((Dibenzofurans OR Fungicides, Industrial[Pharmacological Action] OR acetamides OR furans OR insecticides OR Occupational Diseases OR phenols OR Polyvinyl Chloride OR Vinyl Chloride))))) AND (Humans[Mesh])) AND (Humans[Mesh])​ |
| --- | --- |
| Scopus | ( ( sarcoma  OR  osteosarcoma  OR  "soft tissue sarcoma"  OR  "soft tissue neoplasm" )AND  ( "Environmental Exposure"  OR  "Paternal Exposure"  OR  "Maternal Exposure"  OR  "Inhalation Exposure"  OR  "chemical exposure"  OR  "occupational exposure"  OR  carcinogen  OR  pollutant )  AND  ( ( "Agricultural Worker? Disease?"  OR  herbicide  OR  insecticide  OR  pesticide  OR  "PesticideResidue"  OR  fungicide  OR  defoliant  OR  agrochemical  OR "Occupational Exposure"  OR  "OccupationalDisease")  OR  (acetamide  OR   benzofuran  OR  chlorophenol  OR  dibenzofuran  OR  dioxin  OR  furan  OR  "Hydrocarbons, Chlorinated"  OR  pentachlorophenol  OR  phenol )  OR  ( "Polyvinyl Chloride"  OR  "Vinyl Chloride" )  OR  ( "2,4,5-Trichlorophenoxyacetic Acid"  OR  "2,4-Dichlorophenoxyacetic Acid"  OR  "Agent Orange" ) ) )  AND  ( ( etiology ) )  AND  ( epidemiology )  AND  ( LIMIT-TO ( EXACTKEYWORD ,  "Human" )  OR  LIMIT-TO ( EXACTKEYWORD ,  "Humans" ) )​ |
| Embase | ((sarcoma:ti,ab,kw OR osteosarcoma:ti,ab,kw OR 'soft tissue sarcoma':ti,ab,kw OR 'soft tissue tumor':ti,ab,kw) AND ('environmental exposure':ti,ab,kw OR 'chemical exposure':ti,ab,kw OR 'occupational exposure':ti,ab,kw OR carcinogen:ti,ab,kw OR pollutant:ti,ab,kw) AND ('agricultural worker':ti,ab,kw AND diseases:ti,ab,kw OR herbicide:ti,ab,kw OR insecticide:ti,ab,kw OR pesticide:ti,ab,kw OR 'pesticide residue':ti,ab,kw OR fungicide:ti,ab,kw OR 'defoliant agent':ti,ab,kw OR 'agricultural chemical':ti,ab,kw OR 'occupational disease':ti,ab,kw) OR acetamide:ti,ab,kw OR chlorophenol:ti,ab,kw OR dibenzofuran:ti,ab,kw OR dioxin:ti,ab,kw OR furan:ti,ab,kw OR 'chlorinated hydrocarbon':ti,ab,kw OR pentachlorophenol:ti,ab,kw OR phenol:ti,ab,kw OR plutonium:ti,ab,kw OR radon:ti,ab,kw OR polyvinylchloride:ti,ab,kw OR 'vinyl chloride':ti,ab,kw OR '2,4,5 trichlorophenoxyacetic acid':ti,ab,kw OR '2,4 dichlorophenoxyacetic acid':ti,ab,kw OR 'agent orange':ti,ab,kw) AND (etiology:ti,ab,kw OR epidemiology:ti,ab,kw) |
| Cochrane datebase | sarcoma OR osteosarcoma OR "soft tissue sarcoma" OR "soft tissue neosplasm" in Title Abstract Keyword AND "environmental exposure" OR "inhalation exposure" OR "chemical exposure" OR "occupational exposure" OR carcinogen OR pollutant in Title Abstract Keyword AND "Agricultural Worker? Disease?" OR herbicide OR insecticide OR pesticide OR "pesticide residue" OR fungicide OR defoliant OR agrochemical OR "occupational disease" in Title Abstract Keyword OR acetamide OR arsenic OR arsenical OR benzene OR benzofuran OR chlorophenol OR phenol OR plutonium OR radon in Title Abstract Keyword OR "polyvinyl chloride" OR "vinyl chloride" OR "2,4,5-Trichlorophenoxyacetic acid" OR "2,4-Dichlorophenoxyacetic acid" OR "Agent Orange" AND (etiology OR epidemiology) AND human in Title Abstract Keyword - (Word variations have been searched) |

**Supplementary table 2.** Findings of case-control studies of phenoxy herbicides and chlorophenols

| Reference | Exposure Type | Exposure Assessment | Odds Ration (OR) / Relative Risk (RR) | 95% Confidence interval (CI) |
| --- | --- | --- | --- | --- |
| Hardell et al. 1979 [22] | Occupational exposure to phenoxy acetic acids or chlorophenols | Questionnaire supplemented by telephone interview & employers’ questionnaire to verify employment and the use of chemicals. | RR = 5.7 | 2.9, 11.3 |
| Eriksson et al. 1981 [23] | Occupational exposure to phenoxy acetic acids or chlorophenols | Questionnaire supplemented by telephone interview. | Phenoxy herbicides RR=6.8  Chlorophenols RR = 3.3 | 2.6, 17.3  1.3, 8.1 |
| Greenwald et al. 1984 [24] | Agent orange | Telephone interview or in-person interview. | Service in Vietnam: OR=0.53 | 0.21, 1.3 |
| Smith et al. 1984 [25] | Potential occupational exposure to phenoxy herbicides & chlorophenols | Telephone interviews of subject of next of kin conducted blind by a single interviewer. | OR=1.3 | 0.7, 2.5 |
| Hoar et al. 1986 [26] | Farm herbicide use | Telephone interview & questions on farming practices. | OR=0.9 | 0.5, 1.6 |
| Kang et al. 1986 [27] | Agent orange | Patients’ military personnel records archived at the National Personnel Records Center in St Louis, Missouri. | OR=0.83 | 0.63, 1.09 |
| Kang et al. 1987 [28] | Agent orange | Telephone interview to elicit occupational exposure, medical history, lifestyle and socio-demographic factors. | Vietnam veterans in general: OR=0.85  Army veterans: OR=0.61  Combat veterans: OR=1.06  Combat related MOSC: OR=2.57  Combat in military unit III, were Agent Orang was excessive: OR = 8.64 | 0.54, 1.36  0.32, 1.13  0.42, 2.59  0.72, 9.36  0.77, 111.84 |
| Vineis et al. 1987 [29] | Phenoxy herbicides exposure in rice weeders, assessed by two blinded pesticide researchers. | Direct interview or postal questionnaire, job recorded. | Women: RR=2.7  Women < 75 years old exposed in 1950-1955: RR=15.5 | 0.59, 12.37 |
| Woods et al. 1987 [30] | Occupational exposure to phenoxy acetic acid herbicides & chlorophenols. | Personal interviewes & questionnaires. | Past chlorophenol exposure: OR=0.99  High exposure (Herbicide applicator): OR=1.77 | 0.7, 1.5  0.5, 6.6 |
| Wingren et al. 1990 [31] | Chlorinated phenoxy herbicides & chlorophenols | Detailed questions about pesticide exposure, supplemented by telephone interview. | Gardeners: OR=4.1  Unspecified chemical workers with potential exposure to phenoxy herbicides and/or chlorophenols OR=1.6 | 1.0, 14  0.8, 3.3 |
| Eriksson et al. 1990 [32] | Occupational exposure to dioxins | Mailed questionnaire supplemented with telephone interview. | RR=2.43 (dioxin-containing phenoxyacetic acids or chlorophenols) | 1.30, 4.54 |
| Franceschi et al. 1992 [33] | Employment in agriculture, exposure to herbicides and/or pesticides | Questionnaires | > 10 years employment in agriculture: OR=0.8  > 10 years exposure to pesticides or herbicides OR=0.4  < 10 years exposure to pesticides or herbicides: OR=0.7 | 0.4, 1.5  0.1, 1.2  0.2, 2.5 |
| Smith et al. 1992 [34] | Chlorinated phenoxy compounds or chlorophenols | Personal interviews conducted by an occupational hygienist. | Exposure >1 day: RR=1.0  Exposure > 30 days: RR=2.0 | 0.3, 3.1  0.5, 8.0 |
| Kogevinas et al. 1995 [35] | Workers exposed to any phenoxy herbicides, chlorophenols and dioxins | Individual job records, detailed company questionnaire & company reports. | Exposure to any phenoxy herbicides OR=10.3 | 1.2, 91 |
| Hoppin et al. 1998 [36] | Exposure to chlorophenols, wood preservation (17% jobs) and cutting oils (82% jobs) | Telephone interview by using a standardized questionnaire administered by trained personnel. | High-intensity chlorophenol exposure: OR=1.79  > 10 years of substantial exposure OR=7.78 | 1.10, 2.88  2.46, 24.65 |
| Pahwa et al. 2011 [37] | Phenoxy herbicides | Postal questionnaire & telephone interview for those reporting pesticides exposure of =/> 10 hours per year. | Any phenoxy herbicides OR=1.07 | 0.8, 1.44 |
| Coggon et al. 2015 [38] | Spraying business or factory employment in manufacture of phenoxy herbicide | Personnel, plant records, job histories, death certificates. | >/= 1 year in potentially exposed jobs OR=1.30 | 0.30, 5.62 |

**Supplementary table 3**. Findings of cohort studies of phenoxy herbicides and chlorophenols

| Author/  Year | Exposure type | Exposure Assessment | Case observed / Expected | Outcome | Relative Risk (RR) / Standardized Mortality Ratio (SMR) / Standardized Incidence Ratio (SIR) | 95% Confidence interval (CI) |
| --- | --- | --- | --- | --- | --- | --- |
| Wiklund et al. 1986 [39] | Agricultural or forestry workers | Assumed Occupational exposure to phenoxy acids, no information on individual exposure. | 331/1 508 | Cancer incidence | RR=0.9 | 0.8, 1.0 |
| Wiklund et al.1988 [40] | Licensed pesticide applicators | Estimated exposure | 7 / 7.7 | Cancer incidence | SIR=0.9 | 0.4, 1.9 |
| Saracci et al. 1991 [41] | Workers in production or spraying of phenoxy herbicides | Questionnaires, industry & production records, job histories. | 4 / 2/04 | Mortality | SMR=196  10-19 years from first exposure: SMR=606  In sprayers: SMR=882 | 53, 502  165, 1552  182, 2579 |
| Sathiakumar et al. 1992 [42] | Agricultural chemicals, not including phenoxy herbicides | Plant personal records, death certificates. | 3 / 0/62 | Mortality | - | - |
| Wiklund et al. 1994 [43] | Women employed in agriculture | Population and housing census. | 21 | Cancer incidence in women | SIR=0.62 | 0.39, 0.95 |
| Gambini et al. 1997 [44] | Rice growers | Estimated exposure | 1 / 0.25 | Mortality | SMR=397.3 | 5.2, 2 225.5 |
| Lynge 1998 [45] | Workers in phenoxy herbicides production | Company records | 4 / 2.47 | Cancer Incidence | SIR=1.62 | 0.4, 4.1 |
| Fleming et al. 1999 [46] | Exposure to pesticide in pesticide applicators | Database of pesticide applicators licensed in the State of Florida by the Florida Department of Agriculture and Consumer Services. | Male: 0 / 0.31  Female: 0 / 0.65 | Mortality | Male SMR=0.31  Female SMR=1.74 | 0.0, 1.71  0.01, 16.26 |
| Alavanja et al. 2005 [50] | Pesticide applicators and their wives | Questionnaire | 10 / 15.2 | Cancer incidence | SIR=0.65 (private applicators) | 0.31, 1.20 |
| Coggon et al. 2015 [38] | Phenoxy herbicides | Personnel, plant records, job histories, death certificates. | 4 / 3.3 | Mortality | OR=1.30 (=/>1 year in jobs with potential exposure) | 0.30, 5.62 |

**Supplementary table 4.** Findings of cohort studies for occupational exposures to dioxins

| Author/ Year | Exposure Assessment | Outcome | Number of Cases | Standardized Mortality Ratio (SMR) | 95% Confidence Interval (CI) |
| --- | --- | --- | --- | --- | --- |
| Kogevinas et al. 1997 [51] | Job records, exposure questionnaires, and serum and adipose tissue dioxin levels. | Mortality | Soft tissue sarcoma (STS) (n=6) | SMR=2.03 | 0.75, 4.43 |
| Steenland et al. 1999 [52] | Job descriptions, measuring 2,3,7,8-Tetrachlorodibenzo-p-dioxin (TCDD) in serum. | Mortality | Connective & soft tissue caner (n=4) | SMR=2.32 | 0.63, 5.93 |
| Bodner et al. 2003 [53] | Job records | Mortality | STS (n=2) | SMR=2.4 | 0.3, 8.6 |
| Collins et al. 2009 [54] | Serum dioxin level, work history records, industrial hygiene monitoring data. | Mortality | STS (n=4) | SMR=4.1 | 1.1, 10.5 |

**Supplementary table 5.** Findings of cohort studies of exposures to vinyl chloride monomers

| Author/Year | Person years | Outcome | No of Cases Observed /Expected | Relative Risk (RR)/ Standardized Mortality Ratio (SMR) | 95% Confidence Interval (CI) |
| --- | --- | --- | --- | --- | --- |
| Smulevich et al. 988 [55] | 43216 | Mortality | Angiosarcoma of the liver (ASL) (n=0)  Other soft tissue sarcoma (STS) (n=1) | SMR=1.43 | 0.02, 7.95 |
| Ward et al. 2001 [56] | 324706 | Mortality | ASL (n=37)  Other STS (n=6) | RR=2.9  SMR=1.89 | 2.2, 3.9  0.69, 4.11 |
| Wong et al. 2002 [57] | 40557 | Mortality | ASL (n=0) |  |  |
| Mundt et al. 2017 [58] | 401524 | Mortality | ASL (n= 63)  Other STS (n=1 2 / 4.4) | RR=36.3  SMR=2.43 | 13.1,100.5  1.48, 3.75 |
| Fedeli et al. 2019 [59] | 61736 | Mortality | ASL (n= 9)  Other STS (n= 2) | RR=91.1  SMR=1.61 | 16.8, 497  0.41, 6.50 |

**Supplementary table 6**. Findings of cohort studies of other occupational exposures

| Author/  Year | Exposure  Type | Exposure  Assessment | Outcome | Number of  Cases  Observed/  Expected | Relative Risk  (RR)/  Standardized  Mortality Ratio  (SMR) | 95%  Confidence  Interval (CI) |
| --- | --- | --- | --- | --- | --- | --- |
| Polednak et al. 1978 [66] | Radium dial painting industry | Death certificates; gamma radiation measurements of radon by spectrometry; body burden was estimated from skeletal remains. | Mortality | Bone cancer:  N = 22 / 0.3 | - | - |
| Teta et al. 1988 [67] | Welding fumes, cutting oils, asbestos, organic solvents, environmental ionizing radiation | Company records, files of the Social Security Administration, National Death Index, files of New York State licensed drivers. | Mortality | Connective and other soft tissue cancer: n= 7/1.2 | SMR=583 | 235, 1.202 |
| Wiggs et al. 1994 [68] | Los Alamos National Laboratory employees | Personnel records of Los Alamos National Laboratory and its predecessors. | Mortality | Osteosarcoma: n=1 | - | - |
| Rix et al. 1997 [70] | Sulfite pulp workers | Company records, pension schemes, the National Mortality register; cancer cases obtained from the Danish Cancer Registry. | Cancer incidence | Soft tissue sarcoma (STS) among men: n= 4 / 1.69 | SIR=2.37 | 0.64, 6.06 |
| Rix et al. 1998 [69] | Paper mill workers | Personal cards, company records; cancer cases obtained from the Danish Cancer Registry. | Cancer incidence | STS in women: n= 2 / 1.1  Women employed in paper sorting/ packaging | SIR=1.80  SIR=3.98 | 0.20, 6.51  1.71, 7.84 |
| Koshurnikova et al. 2000 [71] | Exposure to internally deposited plutonium, external gamma radiation | Urine plutonium measurement, film badge monitoring data. | Mortality | STS n=24 /17 | Russian SMR=1.8  U.S. SMR=3.1 | 1.2, 1.6  2.0, 4.6 |

**Supplementary table 7**. Findings of case-control studies of other occupational exposures

| Reference | Exposure Type | Exposure Assessment | Odds ration (OR) / Relative Risk  (RR) | 95% CI |
| --- | --- | --- | --- | --- |
| Balarajan et al. 984 [80] | Farmers and agricultural workers and related occupational groups | The National Cancer Register maintained by the Office of Population Censuses and Surveys. | Farmers, farm managers & market gardener: RR=1.7 | 1.00, 2.88 |
| Hoar et al. 1988 [72] | Insecticide exposure | Telephone interview & questions on farming practices. | OR=1.9 (farmers who failed using PPE while mixing and applying insecticides on animals)  OR=2.0 (farmers who applied insecticides to animals themselves). | 1.1, 3.3  1.1, 3.4 |
| Pearce et al. 1988 [73] | Meat workers | Occupational information was obtained by interviews at the time of hospitalization. | Soft-tissue sarcoma OR=1.90 | 0.90, 4.02 |
| Zahm et al. 1989 [74] | Nonagricultural occupations. | Telephone interview & questions on farming practices. | Woodworking occupation > 6 years: OR=1.2 | 0.9, 3.2 |
| Wingren et al. 1990 [31] | Agricultural and non-agricultural occupations | Mailed questionnaires | Gardeners OR=4.1  Railroad workers OR=3.1  Construction workers with exposure to impregnating agents OR=2.3  Asbestos OR=1.8  Pressure impregnating agents OR=1.7 | 1.0, 14  0.6, 14  0.5, 8.9  0.4, 4.8  0.3, 7.3 |
| Franceschi et al. 1992 [33] | Agricultural and non-agricultural occupations | Histologically confirmed cases who had been admitted as inpatients or referred to outpatient follow up of cancer center in hospitals in study area, administered questionnaire. | Agriculture >10 years: OR=0.8  >10 year chemical agent exposure: OR=1.8  >10 year benzene/other solvent exposure: OR=2.2 | 0.4, 1.5  0.7, 4.4  0.9, 5.5 |
| Hoppin et al. 1999 [75] | Cutting oils, farm work, herbicide use, plywood, sawmill work, solvents, wood or saw dust, formaldehyde, meatpacking or processing, | Telephone interview by using a standardized questionnaire administered by trained personnel. | Herbicide use and Malignant fibrous histiocytoma (MFH): OR=2.94  Meatpacking plant and Dermatofibrosarcoma protuberans (DFSP): OR =2.71  Formaldehyde OR=2.58  Wood or saw dust OR=1.67 | 1.07, 7.32  0.91, 5.33  0.91, 6.39  0.84, 3.24 |
| Briggs et al. 2003 [76] | Various occupations | Telephone and in person questionnaires/ interviews. | Wood dust and STS in African American men OR=3.7 | 1.6, 8.6 |
| Pahwa et al. 2003 [77] | Exposure to farm animals | Self-administered a postal questionnaire. | Chicken farming OR=1.63  Insecticides OR=1.26  Herbicides 0.90 | 1.05, 2.52  0.68, 2.36  0.56, 1.45 |
| Merletti et al. 2006 [78] | Various occupations | Standardized questionnaire was applied by a face-to-face or telephone interviews. | Blacksmiths, toolmakers, machine-tool operators OR =2.14  Woodworkers (especially carpenters) OR=2.68  Construction workers OR=1.62  Ever use of pesticides OR=2.33 | 1.08, 4.26  1.36, 5.29  0.92, 2.87  1.31, 4.13 |
| Hossain et al. 2007 [79] | Agricultural practices, farms, chemical industry, general offices. | Postal questionnaires; provincial cancer registries. | Radium exposure OR=2.78  Temporary jobs on chicken farms OR=1.63  Temporary jobs at apartment complexes OR=2.18 | 1.80, 7.26  1.11, 2.38  1.12, 4.24 |
| Pahwa et al. 2011 [37] | Insecticides | Postal questionnaire & telephone interview for those reporting pesticides exposure of =/> 10 hours per year. | Aldrin OR=3.71  Diazinon OR=3.31 | 1.00, 13.71  1.78, 6.23 |

**Supplementary table 8. PRISMA checklist**

| **Section/topic** | **#** | **Checklist item** | **Reported on page #** |  |
| --- | --- | --- | --- | --- |
| **TITLE** | | |  |  |
| Title | 1 | Identify the report as a systematic review, meta-analysis, or both. | 1 |  |
| **ABSTRACT** | | |  |  |
| Structured summary | 2 | Provide a structured summary including, as applicable: background; objectives; data sources; study eligibility criteria, participants, and interventions; study appraisal and synthesis methods; results; limitations; conclusions and implications of key findings; systematic review registration number. | 2 |  |
| **INTRODUCTION** | | |  |  |
| Rationale | 3 | Describe the rationale for the review in the context of what is already known. | 3-4 |  |
| Objectives | 4 | Provide an explicit statement of questions being addressed with reference to participants, interventions, comparisons, outcomes, and study design (PICOS). | 5 |  |
| **METHODS** | | |  |  |
| Protocol and registration | 5 | Indicate if a review protocol exists, if and where it can be accessed (e.g., Web address), and, if available, provide registration information including registration number. | 5 |  |
| Eligibility criteria | 6 | Specify study characteristics (e.g., PICOS, length of follow-up) and report characteristics (e.g., years considered, language, publication status) used as criteria for eligibility, giving rationale. | 6 |  |
| Information sources | 7 | Describe all information sources (e.g., databases with dates of coverage, contact with study authors to identify additional studies) in the search and date last searched. | 5 |  |
| Search | 8 | Present full electronic search strategy for at least one database, including any limits used, such that it could be repeated. | 5 and Supplementary Table 1 |  |
| Study selection | 9 | State the process for selecting studies (i.e., screening, eligibility, included in systematic review, and, if applicable, included in the meta-analysis). | 5-6 |  |
| Data collection process | 10 | Describe method of data extraction from reports (e.g., piloted forms, independently, in duplicate) and any processes for obtaining and confirming data from investigators. | 6 |  |
| Data items | 11 | List and define all variables for which data were sought (e.g., PICOS, funding sources) and any assumptions and simplifications made. | 6-7 |  |
| Risk of bias in individual studies | 12 | Describe methods used for assessing risk of bias of individual studies (including specification of whether this was done at the study or outcome level), and how this information is to be used in any data synthesis. | 7 |  |
| Summary measures | 13 | State the principal summary measures (e.g., risk ratio, difference in means). | 7 |  |
| Synthesis of results | 14 | Describe the methods of handling data and combining results of studies, if done, including measures of consistency (e.g., I^2^) for each meta-analysis. | 7 |  |
| Risk of bias across studies | 15 | Specify any assessment of risk of bias that may affect the cumulative evidence (e.g., publication bias, selective reporting within studies). | 7 and Supplementary Table 9 |  |
| Additional analyses | 16 | Describe methods of additional analyses (e.g., sensitivity or subgroup analyses, meta-regression), if done, indicating which were pre-specified. | - |  |
| **RESULTS** | | |  |  |
| Study selection | 17 | Give numbers of studies screened, assessed for eligibility, and included in the review, with reasons for exclusions at each stage, ideally with a flow diagram. | 8 |  |
| Study characteristics | 18 | For each study, present characteristics for which data were extracted (e.g., study size, PICOS, follow-up period) and provide the citations. | 8-15 and  Tables 2-7 |  |
| Risk of bias within studies | 19 | Present data on risk of bias of each study and, if available, any outcome level assessment (see item 12). | - |  |
| Results of individual studies | 20 | For all outcomes considered (benefits or harms), present, for each study: (a) simple summary data for each intervention group (b) effect estimates and confidence intervals, ideally with a forest plot. | Supplementary Tables 2-7 |  |
| Synthesis of results | 21 | Present results of each meta-analysis done, including confidence intervals and measures of consistency. | 16-20 and Figures 2-8 |  |
| Risk of bias across studies | 22 | Present results of any assessment of risk of bias across studies (see Item 15). | 21-23 and Supplementary table 9 |  |
| Additional analysis | 23 | Give results of additional analyses, if done (e.g., sensitivity or subgroup analyses, meta-regression [see Item 16]). | - |  |
| Results of individual studies | 20 | For all outcomes considered (benefits or harms), present, for each study: (a) simple summary data for each intervention group (b) effect estimates and confidence intervals, ideally with a forest plot. | Figures 2-8. |  |
| Synthesis of results | 21 | Present results of each meta-analysis done, including confidence intervals and measures of consistency. | 16-20 |  |
| Risk of bias across studies | 22 | Present results of any assessment of risk of bias across studies (see Item 15). | 21-23 |  |
| Additional analysis | 23 | Give results of additional analyses, if done (e.g., sensitivity or subgroup analyses, meta-regression [see Item 16]). | - |  |
| **DISCUSSION** | | | |  |
| Summary of evidence | | 24 | Summarize the main findings including the strength of evidence for each main outcome;  consider their relevance to key groups (e.g., healthcare providers, users, and policy  makers). | 20-23 |
| Limitations | | 25 | Discuss limitations at study and outcome level (e.g., risk of bias), and at review-level (e.g., incomplete retrieval of identified research, reporting bias). | 21-22 |
| Conclusions | | 26 | Provide a general interpretation of the results in the context of other evidence, and  implications for future research. | 24 |
| **FUNDING** | | | |  |
| Funding | | 27 | Describe sources of funding for the systematic review and other support (e.g., supply  of data); role of funders for the systematic review. | None |

*From:*  Moher D, Liberati A, Tetzlaff J, Altman DG, The PRISMA Group (2009). Preferred Reporting Items for Systematic Reviews and Meta-Analyses: The PRISMA Statement.

PLoS Med 6(7): e1000097. doi:10.1371/journal.pmed1000097

**Supplementary Table 9**. Quality assessment of the included studies according to Newcastle-Ottawa scale

| **Case-control Studies** | **Selection** | **Comparability** | **Exposure** | **Quality** |
| --- | --- | --- | --- | --- |
| Hardell at al. 1979 [22] | * * * | * | * * * | 7 |
| Eriksson et al. 1981[23] | * * * | * | * * * | 7 |
| Greenwald et al. 1984 [24] | * * * | * | * * * | 7 |
| Smith et al. 1984 [25] | * * | * | * * * | 6 |
| Hoar et al. 1986 [26] | * * * | * | * * * | 7 |
| Kang et al. 1986 [27] | * * * | * | * * | 6 |
| Kang et al. 1987 [28] | * * * | * | * * * | 7 |
| Vineis et al. 1987 [29] | * * * * | * | * * * | 8 |
| Woods et al. 1987 [30] | * * * | * | * * * * | 8 |
| Wingren et al. 1990 [31] | * * | * | * * * | 6 |
| Eriksson et al. 1990[32] | * * * | * | * * * | 7 |
| Franceschi et al. 1992 [33] | * * | * | *** | 6 |
| Smith et al. 1992 [34] | * * | * | * * * | 6 |
| Kogevinas et al. 1995 [35] | * * * * | * | * * * | 8 |
| Hoppin et al. 1998 [36] | * * * * | * | * * * * | 9 |
| Pahwa et al. 2011 [37] | * * * * | * | * * | 7 |
| Coggon et al. 2015 [38] | * * * | * | * * * | 7 |
| Balarajan et al. 1984 [80] | * * | * | * * * | 6 |
| Hoar Zahm et al. 1988 [72] | * * * * | * | * * * | 8 |
| Pearce et al. 1988 [73] | * * * | * | * * * | 7 |
| Zahm et al. 1989 [74] | * * * * | * | * * * | 8 |
| Hoppin et al. 1999 [75] | * * * * | * | * * * | 8 |
| Briggs et al. 2003 [76] | * * * | * | * * * | 7 |
| Pahwa et al. 2003 [77] | * * * | * | * * * | 7 |
| Merletti et al. 2006 [78] | * * * | * | * * * | 7 |
| Hossain et al. 2007 [79] | * * * | * | * * * * | 8 |
|  |  |  |  |  |
| **Cohort Studies** | **Selection** | **Comparability** | **Exposure** | **Quality** |
| Wiklund et al. 1986 [39] | * * * | * | * * | 6 |
| Wiklund, et.al. 1988 [40] | * * * | * | * * | 6 |
| Saracci et al. 1991 [41] | * * * * | * | * * * * | 9 |
| Sathiakumar et al. 1992 [42] | * * * | * | * * * | 7 |
| Wiklund et al. 1994 [43] | * * * | * | * * | 6 |
| Gambini et al. 1997 [44] | * * * | * | * * * | 7 |
| Lynge 1995 [45] | * * * | * | * * * | 7 |
| Fleming et al. 1999 [46] | * * * | * | * * * | 7 |
| Alavanja et al. 2005 [50] | * * * | * | * * | 6 |
| Coggon et al. 2015 [38] | * * * | * | * * * | 7 |
| Kogevinas et al. 1997 [51] | * * * | * | * * * | 7 |
| Steenland et al. 1999 [52] | * * * | * | * * * * | 8 |
| Bodner et al. 2003 [53] | * * * | * | * * * * | 8 |
| Collins et al. 2009 [54] | * * * | * | * * * * | 8 |
| Smulevich et al. 1988 [55] | * * * * | * | * * * | 8 |
| Ward et al. 2001 [56] | * * * * | * | * * * | 8 |
| Wong et al. 2002 [57] | * * * * | * | * * * | 8 |
| Mundt et al. 2017 [58] | * * * * | * | * * * | 8 |
| Fedeli et al. 2019 [59] | * * | * | * * | 5 |
| Teta et al. 1988 [67] | * * * | * | * * * | 7 |
| Wiggs et al. 1994 [68] | * * * | * | * * | 6 |
| Rix et al. 1998 [69] | * * * | * | * * * | 7 |
| Rix et al. 1997 [70] | * * * | * | * * * | 7 |
| Koshurnikova et al. 2000 [71] | * * * | * | * * * | 7 |
